# A Conjugated Polyelectrolyte with Pendant High Dense Short-Alkyl-Chain-Bridged Cationic Ions: Analyte-Induced Light-Up and Label-Free Fluorescent Sensing of Tumor Markers

**DOI:** 10.3390/polym9060227

**Published:** 2017-06-15

**Authors:** Nina Fu, Yijiao Wang, Dan Liu, Caixia Zhang, Shao Su, Biqing Bao, Baomin Zhao, Lianhui Wang

**Affiliations:** Key Laboratory for Organic Electronics and Information Displays & Institute of Advanced Materials (IAM), Jiangsu National Synergetic Innovation Center for Advanced Materials (SICAM), Nanjing University of Posts & Telecommunications, 9 Wenyuan Road, Nanjing 210023, China; iamnnfu@njupt.edu.cn (N.F.); wangyj365@163.com (Y.W.); liudanxka@163.com (D.L.); 17805005390@163.com (C.Z.); iamssu@njupt.edu.cn (S.S.); iambqbao@njupt.edu.cn (B.B.)

**Keywords:** analyte-induced light-up, conjugated polyelectrolyte, label-free sensing, tumor markers

## Abstract

A novel cationic water-soluble conjugated polyelectrolyte (CPE) of polyfluorene that contains 15% fraction of 2,1,3-benzothiadiazole (BT) units (PFC3NBT) has been obtained. PFC3NBT demonstrates intramolecular energy transfer from the fluorene segments to BT sites when negatively charged species (SDS or DNAs) are added, following by a shift in emission color from blue to green, has been developed. The high density of positive charges and pendent short alkyl chains of *N*-propyltrimethylammoniums endow PFC3NBT with high solubility and high fluorescence quantum efficiency of 33.6% in water. The fluorescence emission properties were investigated in the presence of adverse buffer solutions, different surfactants and DNA strands. Interesting fluorescence emission quenching at short wavelength and fluorescence resonance energy transfer (FRET) induced light-on at BT sites were observed and discussed in detail. Very different from previous reports, the fluorescence emission spectra transition happens with an enhancement of integrated fluorescent intensity. The analytes induced a light-up sensing system was studied with a PFC3NBT/SDS complex mode and confirmed with DNA/DNA-FAM sensing systems. More exciting preliminary results on label-free sensing of tumor markers were also reported by investigating the unique fluorescence response to 11 kinds of proteins. These results provide a new insight view for designing CPEs with light-up and label-free features for biomolecular sensing.

## 1. Introduction

Water soluble, fluorescent π-conjugated polyelectrolytes (CPEs), featured with π-conjugated polymers with water-soluble side chains have attracted much attention in the fields of highly sensitive fluorescence-based sensors [1,2,3,4,5,6,7] and organic electronics [8,9,10,11,12]. By taking advantage of electrostatic interactions between oppositely charged CPEs and biomolecules, CPEs have been rapidly incorporated into homogeneous assays for biologically relevant and clinically important targets including proteins, DNA, carbohydrates, and ions [4,13,14,15,16,17,18,19,20]. Most CPE-based sensors adopt fluorescence quenching as the signal readout [4,21], which generally originates from electron transfer between the CPEs and the analytes upon binding. This sensing strategy always suffers a low sensitivity caused by the non-quenched fluorescence emission of CPE sensors [4]. The strategy of fluorescence resonance energy transfer (FRET) [22,23], by utilizing CPEs as light-harvesting molecules to amplify the signal output of the fluorophores attached to the biomolecular probes, is adopted to address this issue. Although effective, these interchain FRET probing systems are structurally more complicated and require more synthetic efforts to furnish the dye-labelled biomolecules.

Alternatively, considerable attention has been paid to pursuing suitable fluorescent probes giving fluorescent signals via interchain energy transferring from electron-donating segments to electron-accepting ones, leading to achieve the label-free FRET sensors [16,24,25,26,27]. Introduction of 2,1,3-benzothiadiazole (BT) components into fluorene-based polymers is a typical example [16,18,24,25,26,27]. For example, a poly(fluorene-alt-phenylene) derivative (PFBx) with a small fraction of BT units was developed for multicolor DNA sensing, where one important feature of PFBTx was that their emission was concentration dependent. The subscript “x” refers to the percentage of phenylene-BT units in the main chain. Blue emission occurred under dilute conditions (or in the absence of opposite charged analytes) while BT emission was observed in more concentrated conditions (or in the presence of opposite charged analytes). The working hypothesis is that aggregation induced by complexation between the CPEs and an oppositely charged DNAs favors interchain FRET from the blue-emiting phenylene-fluorene segments to the green-emitting BT sites, giving rise to fluorescence change from blue to green. Electrostatic interactions are also essential for controlling the average interchain distance and fluorescence emission response toward the analytes with oppositely charged analytes, and thereby the sensitivity and selectively of the assays [4].

On the other hand, despite the usability of aggregation-enhanced FRET for protein sensing, CPEs with donor-acceptor architecture usually suffer from undesired FRET prior to analyte addition, giving rise to low signal-to-noise ratios and narrow detection range. These drawbacks originate from the hydrophobic nature of the aromatic backbones of CPEs that greatly limits their water solubility and subsequently induces self-aggregation in aqueous solution [24,25,26,27]. Although the undesirable self-aggregation-induced FRET could be possibly minimized by attaching highly water-soluble bulky side chains such as peripheral charged amino groups and cationic poly(ethylene glycol), interchain FRET could be simultaneously inhabited, leading to compromised sensitivity. As such, other sensing concepts that make full use of self-aggretation-induced FRET of CPEs remains unrevealed and greatly challenging for protein sensing. To the best of our knowledge, poly(9,9-bis{30-[(*N*,*N*-dimethyl)-*N*-ethylammonium}-propyl]-2,7-fluorene dibromide) (PFC3N), pendant high density short chain cationic ions, showed the most excellent water solubility up to 100 mg mL^−1^ as well as high photoluminescence (PL) quantum yield of 44% in water [28]. The excellent water solubility in combination with the strong interchain electronstatic repulsion suppressed the polymer aggregation and fluorescence quenching. However, few on the fluorescence sensing application with PFC3N type CPEs were reported. Thus, exploration CPE probes based on PFC3N with a donor-acceptor architecture is of particular interest.

**Scheme 1:**
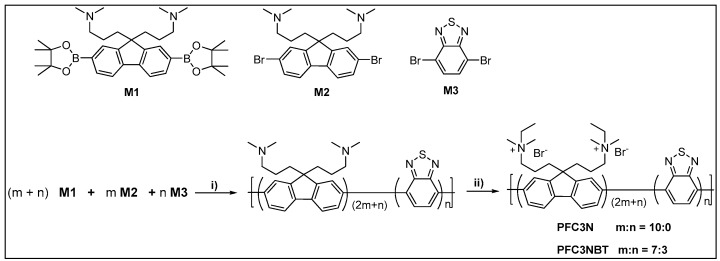
Monomers and synthetic entry into PFC3N and PFC3NBT. (**i**) Pd(PPh_3_)_4_, 2 M K_2_CO_3_, toluene, 80 °C, 24 h ; (**ii**) CH_3_CH_2_Br, dimethyl sulfoxide (DMSO)/water, then acetone.

In this regard, we prepared PFC3NBT, a new derivative of PFC3N, by replacing 15% of its repeating units of fluorene by BT units (as shown in Scheme 1). A BT unit was chosen as the energy acceptor, considering its efficient FRET with polyfluorene segments, as demonstrated in previous reports [16,18,24,25,26,27]. The fluorescence emission behaviors of PFC3NBT in buffer solutions and in the presence of negatively charged analytes were carefully studied. The investigations on the photoluminescence responses of PFC3NBT to SDS, DNA and DNA-FAM reveal that the cationic ions close to the conjugated aromatic backbones played critical roles in the light-up fluorescence sensing by using PFC3NBT. Preliminary results for label-free FRET discriminating tumor markers were also presented.

## 2. Experimental Section

### 2.1. Materials and Instruments

2,7-Bis(4,4,5,5-tetramethyl-1,3,2-dioxaborolan-2-yl)-9,9-bis[30,[30-(*N*,*N*-dimethylamino)-propyl]fluorene (M1) and 2,7-Dibromo-9,9-bis[30,[30-(*N*,*N*-dimethyl-amino)-propyl]-fluorene (M2) were prepared according to the published procedures [28]. Toluene and THF were distilled from sodium in the presence of benzophenone. Other solvents were used as commercial quality. All other chemicals were purchased from Aldrich Shanghai Trading Co Ltd. (Shanghai, China) and Acros Organics (Beijing, China) and used without further purification. The deionized water used in the experiments was obtained from a Modulab 2020 Water purification system (Manufacturer, City, US State abbrev. if applicable, Country). The resistivity, surface tension, and pH of deionized water were 18 MΩ·cm, 72.6 mN/m, and 7.2 at 20.0 ± 0.5 °C, respectively. Tris-HCl buffer solution (10 mM, pH = 6.8) was prepared with Tris base and hydrochloric acid. The aqueous stock solutions of PFC3N and PFC3NBT in deionized (DI) water were prepared and diluted to final concentrations accordingly.

The FAM-labeled and label-free single-stranded DNA sequences were purchased from Sigma and 1st Base. The sequences (5′ to 3′) of all DNAs are as TCT TGA CTA TGT GGG TGC TA and FAM-TCT TGA CTA TGT GGG TGC TA. Bovine serum albumin (BSA) and lysozyme (Lys) were purchased from Sangon Biotech (Shanghai) Co., Ltd. (Shanghai, China). Hemoglobin (Hb), myoglobin (Mb), α-fetoprotein (AFP), carcinoembryonic antigen (CEA), prostate specific antigen (PSA), neuron-specific enolase (NSE), CA19-9, CA125 and CA153 were ordered from Linc-Bio Science Co. Ltd. (Shanghai, China). All solutions were prepared in deionized and sterilized water. The other reagents were of analytical reagent grade and used as purchased without further purification. NMR spectra were recorded on a Bruker Avance 400 MHz spectrometer with tetramethylsilane as the internal standard. Molecular weight and polydispersity of the polymers were determined by gel permeation chromatography (GPC) analysis with polystyrene as standard and THF (HPLC grade) as eluent at a flow rate of 1.0 mL/min. Elemental analysis was performed on a Vario EL III Elementar system (Elementar Analyzen-systeme, Hanau, Germany). Elemental analyses are the average of three runs. The sample was measured simultaneously for C, H, N and S. UV-Vis absorption and photoluminescence (PL) emission spectra were measured using a Shimadzu UV-3150 spectrophotometer and a Shimadzu RF-6301PC spectrophotometer, respectively. The fluorescence quantum yield of the polymers was determined using quinine sulfate (ca. 1 × 10^−5^ M solution in 1.0 M H_2_SO_4_, having the fluorescence quantum yield of 54.6%) as the standard.

### 2.2. Fluorescence Measurements

Analyte-induced aggregation experiments were performed by the successive addition of the aqueous solutions of analytes (SDS, DNAs or other proteins) to the solution of PFC3N or PFC3NBT at 25 °C. For each assay, the analyte solution and the CPE solution were both added in a Quartz cuvette and then shaken for 30 s and the fluorescence spectra were measured after 3 to 5 min [26].

### 2.3. Preparation of Poly({9,9-bis[3’-(N,N-dimethylamino)propyl]-2,7-fluorene-4,7-benzothiadiazole})

A mixture of M1 (588.4 mg, 1.0 mmol), M2 (345.8 mg, 0.70 mmol), M3 (88.0 mg, 0.3 mmol), and Pd(PPh_3_)_4_ (5.5 mg) were added to a 25 mL flask. After the flask was degassed and recharged with nitrogen for three times, the degassed toluene and 2 M potassium carbonate aqueous solution was added via syringe. The mixture was vigorously stirred for 24 h at 80 °C under nitrogen atmosphere. After cooling to room temperature, the mixture was extracted with chloroform, concentrated, and the desired polymer was precipitated from diethyl ether and obtained as a fibrous green solid (462 mg, 63%). ^1^H NMR (400 MHz, CDCl_3_ ): δ (ppm) 7.83–7.82 (br), 7.70–7.68 (br), 2.23–2.05 (br m), 1.03–0.92 (m). ^13^C NMR (100 MHz, CDCl_3_): δ (ppm) 153.2, 151.2, 141.1, 140.2, 126.5, 121.3, 120.3, 60.2, 54.3, 45.4, 36.9, 21.4. Anal. Calcd (%) for C_40.9_H_53.6_N_4_S_0.3_: C, 80.41; H, 8.84; N, 9.17; S, 1.57. Found: C, 80.37; H, 8.78; N, 9.21; S, 1.64.

### 2.4. Synthesis of Poly(9,9-bis{3’-[(N,N-dimethyl)-N-ethylammonium}-propyl]-2,7-fluorene Dibromide)

A 250-mL flask with a magnetic stirring bar was charged with poly({9,9-bis[3’-(*N*,*N*-dimethylamino)propyl]-2,7-fluorene-4,7-benzothiadiazole}) (60.0 mg) dissolved in 10 mL of DMSO. To this solution was added bromoethane (5 mL) and another 15 mL of DMSO. When some precipitates appeared, some water was added to dissolve them. After the reaction mixture was stirred at 45 °C for 72 h, the target polymer was precipitated by the addition of 90 mL of acetone, collected by centrifugation, washed with chloroform and acetone, and dried overnight in a vacuum at 50 °C. The desired polymer was obtained as a yellow to brown solid. ^1^H NMR (400 MHz, D_2_O): δ (ppm) 8.09–7.64 (br), 2.97 (br), 2.66 (br), 2.55 (s), 2.44 (br), 1.09 (br), ^13^C NMR (100 MHz, D_2_O): δ (ppm) 152.9, 150.1, 140.8, 140.5, 127.6, 122.0, 121.3, 62.8, 59.5, 57.6, 55.0, 49.6, 42.4, 36.7, 35.4, 21.4, 17.4, 16.7.

## 3. Results and Discussion

### 3.1. Design Principle of PFC3NBT

The primary concerns for polymer design were to increase substantially the water solubility and suitable ratio of electron-accepting BT units along the conjugated backbone. With this consideration in mind, we chose to synthesize copolymer PFC3N and PFC3NBT, as shown in Scheme 1. As described in previous reports [16,26], if the acceptor fraction BT units was less than 10%, which would result in a low FRET efficiency from polyfluorene segments to BT sites, while, when the fraction of BT units was larger than 20%, the PL spectra of CPEs would display a broad tail from 500 to 650 nm [16]. In the end, a fraction of 15% was chosen in this contribution. Thus, the difference between PFC3N and PFC3NBT is that 15% of the fluorene segments in PFC3N is replaced by BT units randomly to furnish PFC3NBT. The BT content in PFC3NBT was regulated by the ratio of M1, M2 and M3 at the synthesis stage. Since the monomer reactivity ratio of BT and M1 are ambiguous for this type of polymerization, we assume a random distribution of fluorene and BT units throughout the chains. Analysis of the neutral polymers and the targeting quaternized polymers by ^1^H NMR spectroscopy in CDCl_3_ and in D_2_O, respectively, reveals broad overlapping signals that preclude accurate determination of the chemical structures of all polymers. We have determined the number-average molecular weight (*M*_n_) of the neutral precursor of PFC3NBT by GPC analysis, which is ca. 19 kd with a polydispersity index (PDI) = 2.13. This is a moderate molecular weight for CPEs in comparison with the molecular weights of CPEs reported previously. The number-average polymerization degree is estimated to be 62, indicating three BT units in each polymer chain. When heating PFC3NBT, it may generate volatile bromine, and the neutral precursor of PFC3NBT was subjected to EA measurement. Interestingly, the measured content of S is slightly higher than the one calculated according to 15% fraction of BT units. This should be reasonable because, under Suzuki polymerization conditions, the bromine atoms show enhanced reactivity when linking to an electron-deficient BT motif in comparison with linking to fluorene leading to higher fraction of BT in the dynamic polymer chains. For both of the two quaternized polymers, we estimate that PFC3N and PFC3NBT display a water solubility of up to ~60 and 32 mg/mL, respectively, which is higher than most of the known values reported in literature [29,30]. Moreover, the fractional composition of BT in the chains of PFC3NBT can be estimated by measurement of its optical physical properties.

### 3.2. UV-Vis Absorption and Photoluminescence (PL) Spectra

**Figure 1:**
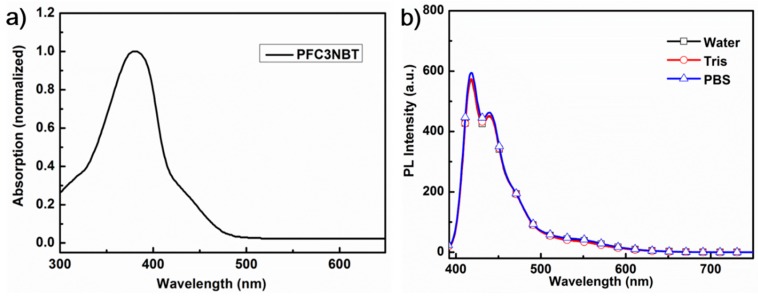
Normalized UV-Vis absorption spectrum of PFC3NBT in water (**a**) and PL spectra of PFC3NBT at RU = 4.5 μM in water (black), Tris HCl (red) and PBS, pH = 7.4 (blue); (**b**) (excitation at 380 nm).

To provide baseline understanding of the intrinsic optical properties of the new CPE of PFC3NBT, it is necessary to compare its optical changes from PFC3N reported previously [22]. Both of the two CPEs have excellent water solubility, thus all of these experiments were carried out in deionized (DI) water without any surfactants or polar solvents [31]. The absorption and photoluminescence (PL) spectra of PFC3NBT were shown in Figure 1. The concentration of PFC3NBT was provided in terms of repeating units (RU). The RUs in Figure 1a,b was 4.5 μM both for absorption spectrum and PL spectra. At this low concentration, the polymer chains are considered in their isolated state. Thus, the profiles display the intrinsic optical properties of polymer chains. The absorption spectrum of PFC3NBT exhibits absorption maximum centered at 382 nm corresponding to fluorene segments and a very weak absorption tail (Figure 1a). The weak absorption tail of PFC3NBT ranging from 420 to 490 nm is assigned to the intermolecular charge transition (ICT) from electron-donating fluorene segments to electron-accepting BT units. By comparison of the intensity of the ICT bands shown in Figure 1a with the ones in the literature, where the BT fractional composition in the chains were from 10 to 20%, one can estimate that the BT fraction in PFC3NBT should be less than 20% but more than 10% [16]. Thus, although the accurate fraction of BT in PFC3NBT chains was not calculated, it should be consistent with the ratio regulated in polymerization stage. According to the results reported by Bazan [16] and us [26], one can speculate that PFC3NBT should share the similar optical property to display a fluorescence emission transition from blue to green when at high CPE concentrations or at the aggregation state formed by the induction of negatively charged analytes.

Interestingly, upon excitation at 380 nm, the PL spectrum of PFC3NBT displays two major emission peaks at 420 and 447 nm, respectively, which is a very similar as PFC3N [28]. The fluorescence quantum yield of PFC3NBT is 33.6%, which is slightly lower than that for PFC3N (43% in our hand), due to the existence of intramolecular energy transfer caused by the BT sites among the polymer backbones. Note that disappearance of the BT emission centered at 545 nm was observed in the PL spectrum of PFC3NBT. This result may be attributed to the low fraction of BT units among the polymer chains and the strong electrostatic repulsion between cationic tetralkylammonium groups pending to the polymer backbones, which suppresses the aggregation of the polymer chains and thus leads to inefficient FRET from fluorene segments to BT sites. Additionally, the relative low fluorescence emitting efficiency of PFC3NBT in pure water is also consistent with a charge-transfer excited state that increases its non-radiative decay rate in more polar solvents. Note that many cationic CPEs were sensitive to ionic strength, which would compromise the reproducibility of the sensing results. We measured the PL spectra of PFC3NBT in Tris-HCl (pH = 6.8) and PBS buffer (pH = 7.4) solutions, two often used buffer solutions for bioassays. Excitingly, no obvious changes of the PL spectra were observed when compared with that in pure DI water (as shown in Figure 1b). This result indicates that PFC3NBT can be used as a CPE probe in multiple buffer conditions without compromising its emission property.

### 3.3. PL Response of PFC3NBT to Sodium Dodecyl Sulfate (SDS)

**Figure 2:**
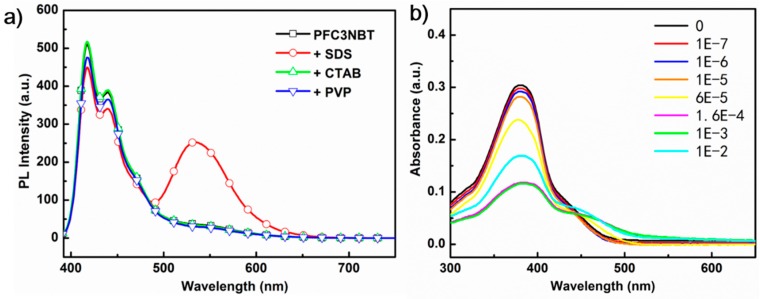
(**a**) PL spectra of PFC3NBT in the absence and presence of surfactant of SDS, CTAB, PVP ((RU) = 4.5 μM, [surfactant] = 5 μM, excitation at 380 nm); (**b**) UV-Vis spetra of PFC3NBT ([RU] = 4.5 μM) in the absence and presence of SDS with the concentration ranging from 0 to 20 mM.

**Figure 3:**
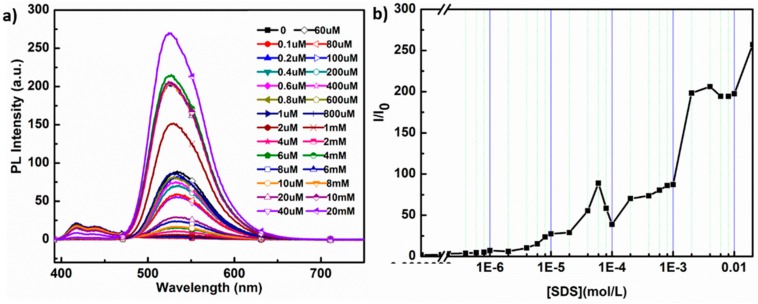
(**a**) PL spectra of PFC3NBT in the absence and presence of SDS with the concentrations of SDS ranging from 0 to 20 *M*_m_; (**b**) fluorescence intensity at 545 nm (*I*_545_) in the prescence of SDS at adverse concentrations vs. that in the absence of SDS ((RU) of PFC3NBT = 4.5 μM, excitation at 380 nm).

The amphiphilicity of CPEs drives the formation of aggregates in presence of species with opposite charge, such as surfactants [32,33,34,35,36,37]. As we mentioned before, the FRET from blue-emitting fluorene segments to green-emitting BT segments of PFC3NBT would be enhanced when interchain contacts exist in aggregates [16,24,25,26]. We investigated the PL responses of PFC3NBT to surfactants including SDS (negatively charged), PVP (neutral polymer surfactant) and CTAB (positively charged). As showed in Figure 2a, the neutral PVP has negligible impact on the PL spectrum of PFC3NBT, while the positively charged surfactant of CTAB slightly diminishes the fluorescence emission intensity. Instead, the addition of negatively charged SDS results in dramatic changes of the PL spectrum, including a decrease at a shorter wavelength regime and obvious enhancement of BT emission output. In addition, the total integration under the PL curve (the red line shown in Figure 3a) increased dramatically. Fluorescence intensity at 545 nm (*I*_545_) in the presence of SDS at adverse concentrations vs. that in the absence of SDS (*I*_0_) was recorded in Figure 3b, indicating that concentration-depended FRET enhancement from PF segments to BT sites was improved along with the concentration increase of SDS. This aggregation-induced emission enhancement (AIEE) is unusual for CPEs because most of the reported CPEs underwent an aggregation induced quenching (AIQ). The working hypothesis of FRET from fluorene segments to BT sites can explain the changes of PL spectrum, but it cannot give conclusive explanation on the AIEE phenomenon. In addition, this phenomenon is very different from the results reported by Heeney [32]. As their descriptions, the PL intensity of a cationic polyfluorene derivative, poly(9,9′-bis(6-*N*,*N*,*N*-trimethylammonium-hexyl)fluorene-alt-1,4-phenylene) dibromide (FPQ-BR), was diminished at lower surfactant concentrations, where SDS induced the formation of a cross-linking aggregation. At higher SDS concentrations, the PL intensity of FPQ-BR was increased because the formed SDS micelles can remain electrostatically tethered to FPQ-BR chains or cylindrically encapsulate FPQ-BR to induce the polymer chains extension. Obviously, according to this mechanism, the efficient FRET from polyfluorene segments to BT site would not be observed in this study because the high concentrated SDS limited the interchain contacts.

There should be another driven force accounting for this surfactant induced AIEE phenomena, except for two favorable interactions (electrostatic and hydrophobic interactions) between the CPE and surfactant. Generally, two functions of the pendent cationic tetralkylammonium groups were mentioned, including increasing the water solubility of cationic CPEs and providing the electrostatic interaction with negatively charged analytes (DNAs or proteins). We speculate the mechanism for this phenomenon as follows. SDS features a long hydrophobic chain and head group with negative charge to bind to CPE side chains. With the addition of SDS to CPE solution, the cationic/anionic ion pairs are formed. One can readily imagine that the cationic/anionic ion pair will be surrounded by water, but the long hydrophobic chain will be mixed with the hydrophobic conjugated backbones of CPE [33,34]. With the increase of SDS concentration, there should be more hydrophobic chains of SDS entangling with CPE backbone. Thus, the CPE aggregates will be unraveled and the aggregation induced fluorescence quenching will be suppressed, but the efficient interchain FRET from fluorene segments to BT sites is not affected. Consequently, both SDS induced enhancement of fluorescence emission of BT sites and improved FRET efficiency were observed. Of course, the short alkyl chain in PFC3NBT will make longer hydrophobic alkyl chain of SDS contact with the backbone of PFC3NBT. In addition, when at higher concentrations of SDS, the micelles are formed and the extended backbone will be twisted, which results in the reduction of conjugation length of CPE. We can observe that, at higher SDS concentration, the fluorescence emission of BT sites shows a red shift. Finally, the integrated PL intensity was increased; correspondingly, an unusual AIEE effect was observed. In contrast, the presence of PVP and CTAB does not induce the changes of microenvironment around PFC3NBT, consistent with the PL measurements showing little interaction between these non-complementary materials.

### 3.4. PL Response of PFC3NBT and PFC3N to DNAs

**Figure 4:**
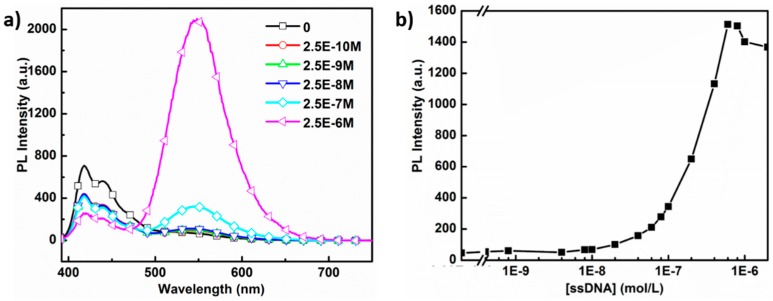
(**a**) PL spectra of PFC3NBT after addition of ssDNA at adverse concentrations; (**b**) fluorescence intensity at 545 nm in the presence of ssDNA at adverse concentrations ((RU) of PFC3NBT = 4.5 μM, excitation at 380 nm).

Having established the idea that changes in PL spectra and integrated intensity of PFC3NBT were driven by the amphiphilicity driving aggregation, we undertook the titration experiments on PFC3NBT using ssDNA. The sensing properties of CPEs to ssDNA and FAM-tagged ssDNA were carried out in de-ionized (DI) water at a polymer concentration of 4.5 μM in RU. As shown in Figure 4a, the PL responses of PFC3NBT to ss-DNA are dependent on ssDNAs. Along with the increase of ssDNA from 0 to 2.5 μM, the fluorescence emission intensity of BT increased dramatically at the expense of fluorescence emission intensity of PF segments, indicating an efficient interchain FRET occurred. The fluorescence peak values of BT sites were recorded in Figure 4b, which displayed maximum at micromole levels of [ssDNA]. One can expect that the fluorescence peak intensity of BT would increase along with the increase of PFC3NBT concentration.

**Figure 5:**
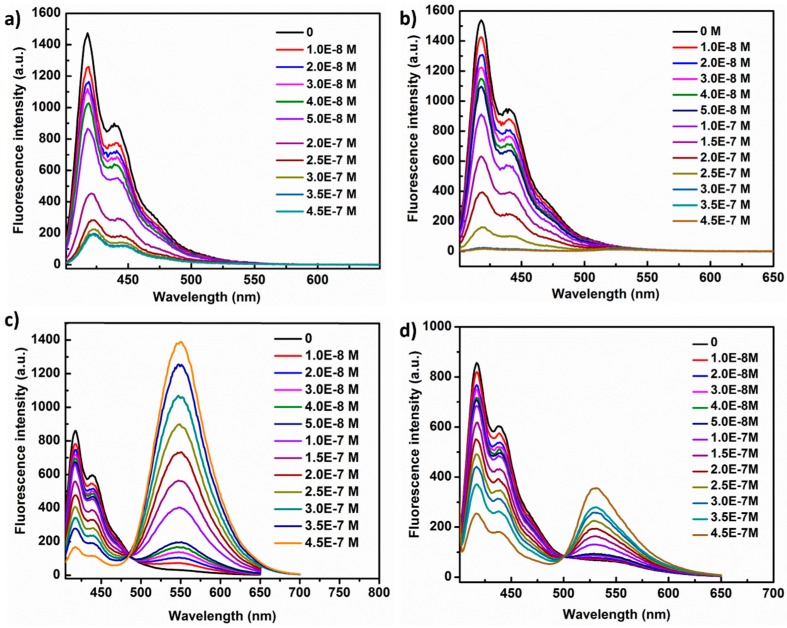
PL spectra of PFC3N after addition of ssDNA (**a**) and ssDNA-FAM (**b**) at adverse concentrations; PL spectra of PFC3NBT after addition of ssDNA (**c**) and ssDNA-FAM (**d**) at adverse concentrations ((RU) of CPEs = 6 μM, excitation at 380 nm).

To confirm the analyte-induced FRET enhancement observed in CPE/SDS titration experiments, we further investigated the similar titration of DNA and FAM-tagged DNA for comparison. As shown in Figure 5a, the PL intensity of PFC3N were quenched by about seven-fold when [ss-DNA] increased from 1.0 × 10^−8^ to 4.5 × 10^−7^ M. Surprisingly, when ss-DNA-FAM was used in the same titration for PFC3N, the changes of PL spectrum showed the same trend and no obvious FRET from PFC3N to FAM was observed (Figure 5b). This can help us advance the idea that negatively charged DNA can only induce the aggregation of PFC3N without unraveling the aggregates of PFC3N backbone, whereas only AIQ of PFC3N was observed. This is consistent with the assumption in the SDS titration section that the hydrophobic alkyl chain of SDS plays an important role in suppressing the AIQ of PFC3NBT. In the case of PFC3NBT, its PL intensity at 418 nm was diminished upon the complexation with ss-DNA; however, simultaneously, the fluorescent emission peak at 545 nm was increased dramatically (see in Figure 5c), indicating an efficient FRET from polyfluorene segment to BT sites. The result was consistent with the report by Bazan [16]. However, upon titration of PFC3NBT with FAM tagged DNA at adverse concentrations, emission peaks at 532 nm were observed and increased in intensity (see Figure 5d). This peak can be considered as the combination of BT emission peak at 545 nm and FAM emission at 518 nm, indicating the formation of polymer/DNA complexes, whereas the BT emission contributes 57.2% of the peak (λ_532_) intensity and FAM emission accounts for another 42.8%. Note that the FRET efficiency from polyfluorene segments to BT sites is lowered, but the FRET efficiency from PFC3NBT to FAM is enhanced. The titration study of PFC3NBT with DNA and SDS can help us establish the knowledge that CPE with better water-solubility and short pendent alkyl chains shows extreme sensitivity to the changes of its microenvironments. In addition, the FRET from PF to BT sites can be applied as probes for label-free detection of analytes.

### 3.5. PL Responses of PFC3NBT to Different Proteins

Proteins are essential biomolecules in living organisms: many proteins are enzymes that catalyze important biochemical reactions, and proteins also perform structural and mechanical functions in maintaining cell shape. Specially, small proteins such as antibodies function as tumor markers. Therefore, sensitive detection of tumor markers is of vital importance for tumor diagnosis and the therapy at its early stage. Motivated by the analytes-mediated FRET between fluorene segments and BT units, a series of important proteins closely related to human health were selected to further investigate their influence on the fluorescence emission of PFC3NBT, in order to pave the way of utilizing it as a kind of the label-free and lighting-up sensing agent for protein detection. The final concentration of PFC3NBT is at 4.5 μM ([RUs]) in phosphate buffered saline (PBS, 2 mM, pH = 7.4). These proteins include BSA, Hb, Lys, Mb, AFP, CEA, PSA, NSE, CA19-9, CA125 and CA153. The details for these proteins and tumor markers are included in Table S1 (see ESI). The PL spectra responses of PFC3NBT toward 11 proteins are recorded upon excitation at 380 nm. Furthermore, the final concentrations of proteins are at 0.25 μM, except for CA19-9 (at 250 ku), CA125 (at 250 ku) and CA153 (at 5 ku).

**Figure 6:**
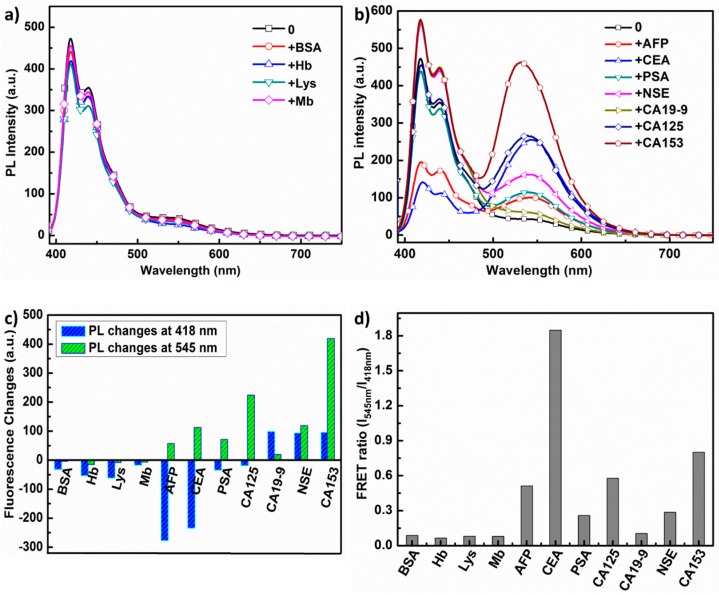
PL spectra of PFC3NBT in PBS (pH = 7.4) in the absence and presence of different proteins (**a**) for BSA, Hb, Lys and Mb; (**b**) for AFP, CEA, PSA, CA125, CA19-9, NSE and CA153. RU = 4.5 μM, [protein] = 0.25 μM or less. Excitation at 380 nm; (**c**) the changes of the fluorescence emission peaks at 418 (∆*I*_418 nm_) and 545 nm (∆*I*_545 nm_) as a function of proteins; (**d**) the FRET ratio of the green emission at 545 nm to the blue emission of PFC3NBT at 418 nm (*I*_545_/*I*_418_) as a function of proteins.

The PL spectra of PFC3NBT/protein mixtures were recorded and displayed in Figure 6a,b. As expected, after adding adverse proteins, the cationic polymer displays distinct PL emission features. For example, BSA, Hb, Lys and Mb have negligible influence on the PL spectra of PFC3NBT (Figure 6a), except for slight decrease of the fluorescence peaks at 418 and 545 nm. Interestingly, none of these four proteins are tumor markers. In contrast to them, the other seven proteins, which are all tumor markers, exhibit significant influence on the PL spectra of PFC3NBT. In the cases of AFP, CEA, PSA and CA125, a conspicuous fluorescence emission intensity increase at 545 nm were displayed at the expense of the fluorescence emission intensity at 418 nm (Figure 6b). There are still some differences observed. When mixed with AFP and CEA, the fluorescence emission at 418 nm was quenched dramatically, but the peak at 545 nm was increased slightly. In the presence of PSA and CA125, the fluorescence emission intensity of the polyflurene segments at 418 nm were slightly quenched at a fraction of 5% and 8% for PSA and CA125, respectively, but the fluorescence emission at 545 nm for BT sites were dramatically increased, especially for CA125. The trend of PFC3NBT fluorescence emission response to NSE, CA19-9 and CA153 is different from other proteins. As shown in Figure 6b, both of the fluorescence emission peaks at 418 nm and 545 nm are increased. In particular, CA153 shows intensive influence on the PL of PFC3NBT at a very low concentration of 5 ku. The changes of the fluorescence emission peaks at 418 (∆*I*_418 nm_) and 545 nm (∆*I*_545 nm_) were calculated and compared in Figure 6c. Clearly, the PL response to 11 proteins can be roughly classified into three classes: (1) both the intensity at 418 and 545 nm were quenched; (2) the PL intensity at 545 nn increased at the expense of the intensity at 418 nm and (3) both the PL intensity at 418 and 545 nm were increased. To quantify the protein effect on the FRET efficiency of PFC3NBT, FRET ratio (*I*_545_/*I*_418_) in the presence of proteins (the concentrations are as mentioned to each protein) is summarized in Figure 6d, which are calculated according to the peak values showed in Figure 6a,b. Obviously, all tumor markers enhanced the FRET efficiency outperforming those non-tumor markers. The experimental results reveal that PL transition trends of PFC3NBT exhibit good selectivity and specificity toward these proteins. Thus, PF3CNBT could be used as a probe to discriminate these tumor makers with the fingerprint features displayed by the PL responses showed in Figure 6c.

The selectively enhanced FRET responses of PFC3NBT should be mainly associated with the polymer/protein interactions including hydrophobic and electrostatic interactions. According to pI (isoelectric point) values, these proteins can be divided into two groups at pH = 7.4: the negatively charged proteins (BSA, Hb, AFP, CEA, PSA and NSE) and the positively charged protein of Lys. Considering the fact that PFC3NBT is positively charged due to the high dense of cationic ions surrounding the backbones, electrostatic attraction should exist for the former group. However, the addition of these oppositely charged proteins into PFC3NBT solution does not contribute to further enhancing the FRET efficiency similarly, as shown in Figure 6a–c. Since the lackness of pI data for CA19-9, CA125 and CA153, the electrostastic interaction with PFC3NBT is unclear. On the other hand, PFC3NBT shows only PF blue emission at 4.5 μM, indicating no self-aggregation forms intrinsically in aqueous solution. The FRET from polyfluorene segments to BT sites should in principle be induced by the formation of polymer/protein complexes, where the interchain contacts formed to allow energy transfer from PF to BT. We speculate that structural conformations and the distribution of surface charges of proteins dominate the aggregation of CPE chains, resulting in this interesting PL response to proteins. When cationic CPE is mixed with these proteins, the CPE chains selectively tether to certain parts of the proteins where the negative charges localized. The protein molecular weight, structural conformation and the localized charges among certain proteins will impart significant influence on the interchain energy transfer among the CPE aggregates, and then the specific PL response to each protein. Obviously, different proteins show comprehensive effects on the interchain FRET from PF to BT. Thus, the CPE shows a specific FRET behavior toward the tumor marker proteins. It should be mentioned that most of the measured proteins that demonstrate an influence on the PL spectra of PFC3NBT are glycoproteins, except for Lys and Mb. Although the fundamentals for this phenomena is still unclear at this stage, we can convey the idea that the unique PL response of PFC3NBT to glycoproteins must be very important for early in-clinic diagnosis of tumor markers [27,38].

**Figure 7:**
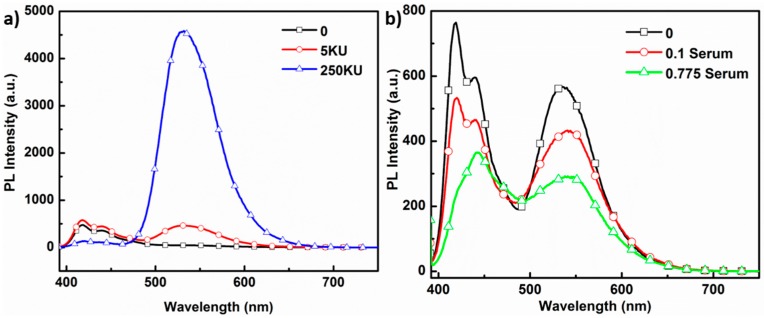
(**a**) PL spectra of PFC3NBT in PBS (pH = 7.4) in the absence and presence of CA153 at the concentration of 0 ku, 5 ku and 250 ku. (**b**) PL spectra of PFC3NBT in PBS (pH = 7.4) in the presence of CA153 = 5 ku with a different fraction of serum in PBS/serum mixture (fraction calculated by *v*/*v*). RU = 4.5 μM. Excitation at 380 nm.

As it was mentioned above, CA153 enhanced the FRET efficiency of PFC3NBT at a very low concentration of 5 ku. We further increased the concentration of CA153 from 0 to 5 to 250 ku and measured the PL spectra of the mixtures. As shown in Figure 7a, the fluorescence emission at 418 nm was increased slightly when [CA153] = 5 ku, and then decreased dramatically when [CA153] = 250 ku, whilst the fluorescence emission intensity at 545 nm increased successively. The corresponding FRET ratio increased from 0.8 to 38.6, indicating that PFC3NBT displayed the best selectivity toward CA153 compared with other proteins at similar concentrations. To establish the idea of the utilization of PFC3NBT as a light-up and label-free probe for these proteins, the fluorescence emission changes were also measured in the presence of serum. As shown in Figure 7b, with the fraction (*v*/*v*) of serum in DI water to DI water increased from 0 to 0.775, both of the two fluorescence emission peaks at 418 and 545 nm were quenched. Interestingly, the normalized profiles (see Figure S1) displayed similar FRET ratios even in the high fraction of serum to water, indicating that PF3CNBT can be used as a promising probe for detection CA153 in serum containing solution. In the end, this experiment indicates the possibility to build-up a fluorescence light-up imaging probe utilizing PFC3NBT in vitro or in vivo imaging.

## 4. Conclusions

We have designed and synthesized a CPE, PFC3NBT, by incorporating polyfluorene with 15% fraction of benzo[2,1,3]thiadiazole (BT) in the conjugated backbones and high dense C3 alkyl chains as the pendant cationic ions. PFC3NBT shows excellent water solubility and blue emission in dilute DI solution with fluorescent emission quantum efficiency of 33.6%. The high density of water-soluble cationic ions bridged by short C3 alkyl chain not only enhance the solution stability of PFC3NBT, but also impart strong influence on its unique photoluminescence response toward different analytes including SDS, ssDNA and proteins. Upon formation of complex with SDS micelles, efficient FRET from PF segment to BT sites and fluorescence light-up were both observed. The mechanism was also discussed. The optical properties of PFC3NBT and PFC3N upon being mixed with ssDNA and FAM-tagged ssDNA in DI water individually are investigated to reveal the fact that the short C3 alkyl bridged cationic ions are critical to the FRET effect from PF segments to BT sites, which simultaneously imparts great influence on the FRET from CPE to FAM dye. According to these results, PFC3NBT is confirmed as an analyte-induced light-up and label-free probe. More importantly, the fluorescence signal change of PFC3NBT toward 11 kinds of proteins displayed very different trends, leading to the idea that PFC3NBT can be used as the probe to discriminate different kinds of tumor markers. PFC3NBT especially holds great promise for rapid detection and quantification of CA153 with higher sensitivity than six other kinds of tumor markers.

In conclusion, this study has demonstrated a new generation of cationic CPEs with donor-acceptor backbones that circumvent the solubility-caused limitation to possess analyte-induced light-up and label-free characteristics toward negatively charged chemicals and biomolecules, especially for tumor marker sensing. From the materials viewpoint, other electron-deficient units can be incorporated into polyfluorene backbone with pendant high density of shorter alkyl-chain-bridged ions to deliver a similar PL response behavior toward analytes. As a result, this study will pave the way to develop CPEs not only for fluorescence sensing applications but also as probes for fluorescence imaging in vitro or in vivo.

## Supplementary Materials

The following are available online at www.mdpi.com/2073-4360/9/6/227/s1, Figure S1: Normalized PL spectra of polymer/CA153 in serum/PBS mixture. [RU] = 4.5 μM, [CA153] = 5 ku. Fraction values calculated by *V*_serum_/(*V*_PBS_ + *V*_serum_). Excitation at 380 nm and normalized at 440 nm. Table S1: Isoelectric point (pI) of each protein used in this work.

## References

[1] Gaylord B.S., Heeger A.J., Bazan G.C. (2002). DNA detection using water-soluble conjugated polymers and peptide nucleic acid probes. Proc. Natl. Acad. Sci. USA.

[2] Thomas S.W., Joly G.D., Swager T.M. (2007). Chemical sensors based on amplifying gluorescent conjugated polymers. Chem. Rev..

[3] Liu B., Bazan G.C. (2004). Interpolyelectrolyte complexes of conjugated copolymers and DNA: Platforms for multicolor biosensors. J. Am. Chem. Soc..

[4] Zhu C.L., Liu L.B., Yang Q., Lv F.T., Wang S. (2012). Water-soluble conjugated polymers for imaging, diagnosis, and therapy. Chem. Rev..

[5] Gaylord B.S., Heeger A.J., Bazan G.C. (2003). DNA hybridization detection with water-soluble conjugated polymers and chromophore-labeled single-stranded DNA. J. Am. Chem. Soc..

[6] Pinto M.R., Schanze K.S. (2004). Amplified fluorescence sensing of protease activity with conjugated polyelectrolytes. Proc. Natl. Acad. Sci. USA.

[7] Pu K., Liu B. (2011). Fluorescent conjugated polyelectrolytes for bioimaging. Adv. Funct. Mater..

[8] He Z., Zhong C., Huang X., Wong W.Y., Wu H., Chen L., Su S., Cao Y. (2011). Simultaneous enhancement of open-circuit voltage, short-circuit current density, and fill factor in polymer solar cells. Adv. Mater..

[9] Zhou Y., Fuentes-Hernandez C., Shim J., Meyer J., Giordano A.J., Li H., Winget P., Papadopoulos T., Cheun H., Kim J. (2012). A universal method to produce low–work function electrodes for organic electronics. Science.

[10] Guan X., Zhang K., Huang F., Bazan G.C., Cao Y. (2012). Amino N-oxide functionalized conjugated polymers and their amino-functionalized precursors: new cathode interlayers for high-performance optoelectronic devices. Adv. Funct. Mater..

[11] Meng B., Fu Y., Xie Z., Liu J., Wang L. (2014). Phosphonate-functionalized donor polymer as an underlying interlayer to improve active layer morphology in polymer solar cells. Macromolecules.

[12] Duarte A., Pu K., Liu B., Bazan G.C. (2011). Recent advances in conjugated polyelectrolytes for emerging optoelectronic applications. Chem. Mater..

[13] Feng F.D., He F., An L.L., Wang S., Li Y.L., Zhu D.B. (2008). Fluorescent conjugated polyelectrolytes for biomacromolecule detection. Adv. Mater..

[14] Liu X.F., Tang Y.L., Wang L.H., Zhang J., Song S.P., Fan C.H., Wang S. (2007). Optical detection of mercury(II) in aqueous solutions by using conjugated polymers and label-free oligonucleotides. Adv. Mater..

[15] Li C., Numata M., Takeuchi M., Shinkai S. (2005). A sensitive colorimetric and fluorescent probe based on a polythiophene derivative for the detection of ATP. Angew. Chem. Int. Ed..

[16] Chi C.Y., Mikhailovsky A., Bazan G.C. (2007). Design of cationic conjugated polyelectrolytes for DNA concentration determination. J. Am. Chem. Soc..

[17] Liu B., Bazan G.C. (2004). Homogeneous fluorescence-based DNA detection with water-soluble conjugated polymers. Chem. Mater..

[18] Zhan R.Y., Liu B. (2015). Benzothiadiazole-containing conjugated polyelectrolytes for biological sensing and imaging. Macromol. Chem. Phys..

[19] Wang S., Gaylord B.S., Bazan G.C. (2004). Fluorescein provides a resonance gate for FRET from conjugated polymers to DNA intercalated dyes. J. Am. Chem. Soc..

[20] Feng F.D., Liu L.B., Wang S. (2010). Fluorescent conjugated polymer-based FRET technique for detection of DNA methylation of cancer cells. Nat. Protoc..

[21] Sun H., Feng F., Yu M.H., Wang S. (2007). Analyte-induced aggregation of a water-soluble conjugated polymer for fluorescent assay of oxalic acid. Macromol. Rapid Commun..

[22] Maynor M.S., Nelson T.L., O’Sullivan C., Lavigne J.J. (2007). A food freshness sensor using the multistate response from analyte-induced aggregation of a cross-reactive poly(thiophene). Org. Lett..

[23] Nelson T.L., O’Sullivan C., Greene N.T., Maynor M.S., Lavigne J.J. (2006). Cross-reactive conjugated polymers: analyte-specific aggregative response for structurally similar diamines. J. Am. Chem. Soc..

[24] Liu B., Bazan G. (2006). Optimization of the molecular orbital energies of conjugated polymers for optical amplification of fluorescent sensors. J. Am. Chem. Soc..

[25] An L.L., Tang Y.L., Feng F.D., He F., Wang S. (2007). Water-soluble conjugated polymers for continuous and sensitive fluorescence assays for phosphatase and peptidase. J. Mater. Chem..

[26] Bao B.Q., Yuwen L.H., Zheng X., Weng L.X., Zhu X.R., Zhan X.W., Wang L.H. (2010). A fluorescent conjugated polymer for trace detection of diamines and biogenic polyamines. J. Mater. Chem..

[27] Sun P.F., Lu X.M., Fan Q.L., Zhang Z.Y., Song W.L., Li B., Huang L., Peng J.W., Huang W. (2011). Water-soluble iridium(III)-containing conjugated polyelectrolytes with weakened energy transfer properties for multicolor protein sensing applications. Macromolecules.

[28] Wang H.P., Lu P., Wang B.L., Qiu S., Liu M.R., Hanif M., Cheng G., Liu S.Y., Ma Y.G. (2007). A water-soluble π-conjugated polymer with up to 100 mg·mL^−1^ solubility. Macromol. Rapid Commun..

[29] Huang F., Hou L.T., Wu H.B., Wang X., Shen H.L., Cao W., Yang W., Cao Y. (2004). High-efficiency, environment-friendly electroluminescent polymers with stable high work function metal as a cathode: Green- and yellow-emitting conjugated polyfluorene polyelectrolytes and their neutral precursors. J. Am. Chem. Soc..

[30] Burrows H.D., Lobo V.M.M., Pina J., Ramos M.L., Seixas de Melo J., Valente A.J., Tapia M.M.J., Pradhan S., Scherf U. (2004). Fluorescence enhancement of the water soluble poly{1,4-phenylene-[9,9-bis(4-phenoxybutylsulfonate)]fluorene-2,7-diyl} copolymer in n-dodecylpentaoxyethylene glycol ether micelles. Macromolecules.

[31] Wang Y., Zhou Z.J., Zhu J.S., Tang Y., Canady T.D., Chi E.Y., Schanze K.S., Whitten D.G. (2011). Dark antimicrobial mechanisms of cationic phenylene ethynylene polymers and oligomers against *Escherichia coli*. Polymers.

[32] Heeley M.E., Gallaher J.K., Nguyen T.L., Woo H.Y., Hodgkiss J.M. (2012). Surfactant controlled aggregation of conjugated polyelectrolytes. Chem. Commun..

[33] Dutta K., Mahale A., Arulkashmir A., Krishnamoorthy K. (2012). Reversible assembly and disassembly of micelles by a polymer that switches between hydrophilic and hydrophobic wettings. Langmuir.

[34] Attar H.A., Monkman A.P. (2007). Effect of surfactant on water-soluble conjugated polymer used in biosensor. J. Phys. Chem. B.

[35] Monteserin M., Burrows H.D., Mallavia R., Di Paolo R.E., Macanita A.L., Tapia M.J. (2010). How to change the aggregation in the DNA/surfactant/cationic conjugated polyelectrolyte system through the order of component addition: anionic versus neutralsurfactants. Langmuir.

[36] Malik A.H., Hussain S., Iyer P.K. (2016). Aggregation-induced FRET via polymer–surfactant complexation: A new strategy for the detection of spermine. Anal. Chem..

[37] Wang S., Zeman C.J., Jiang J., Pan Z., Schanze K.S. (2017). Intercalation of alkynylplatinum(II) terpyridine complexes into a helical poly(phenylene ethynylene) sulfonate: application to protein sensing. ACS Appl. Mater. Interfaces.

[38] Yuan Z.Q., Du Y., Tseng Y.-T., Peng M.H., Cai N., He Y., Chang H.-T., Yeung E.S. (2015). Fluorescent gold nanodots based sensor array for proteins discrimination. Anal. Chem..

