# Supplementary material for: A Conjugated Polyelectrolyte with Pendant High Dense Short-Alkyl-Chain-Bridged Cationic Ions: Analyte-Induced Light-Up and Label-Free Fluorescent Sensing of Tumor Markers
Source: Polymers (Basel). 2017 Jun 15;9(6):227. doi: 10.3390/polym9060227 (PMC6432017; doi:10.3390/polym9060227)

SUPPLEMENTARY MATERIALS for

A Conjugated Polyelectrolyte with Pendant High Dense Short-Alkyl-Chain-Bridged Cationic Ions: Analyte-Induced Light-Up and Label-Free Fluorescent Sensing of Tumor Markers

Nina Fu, Yijiao Wang, Dan Liu, Caixia Zhang, Shao Su, Biqing Bao, Baomin Zhao† and Lianhui Wang†

Key Laboratory for Organic Electronics and Information Displays & Institute of Advanced Materials (IAM), Jiangsu National Synergetic Innovation Center for Advanced Materials (SICAM), Nanjing University of Posts & Telecommunications, 9 Wenyuan Road, Nanjing 210023, China.

† Correspondence: iambmzhao@njupt.edu.cn; iamlhwang@njupt.edu.cn; tel.:+86-25-8586-6332

Table S1. Isoelectric point (pI) of each protein used in this work.

Protein	Full name	pI	Molecualr weight (kDa)	Glyco-protein
BSA	bovine serum albumin	4.9	66	no
Hb	hemoglobin	7.07	64	no
Lys	Lyso zyme	11.0-11.2	14	yes
Mb	myoglobin	6.99	17	yes
AFP	alpha fetoprotein	4.8	7	yes
CEA	carcinoembry onic antigen	4.8	200	yes
PSA	Prostate specific antigen	6.9	33	yes
NSE	neuron-specific enolase	4.7	78	yes
CA19-9	Cancer related antibody	-	36	yes
CA125	Cancer related antibody	-	>5000	yes
CA153	Cancer related antibody	<5	-	yes

Figure S1. Normalized PL spectra of polymer/CA153 in serum/PBS mixture. [RU] = 4.5 μM, [CA153] = 5 ku. Fraction values calculated by $V_{serum}/(V_{PBS}+V_{serum})$. Excitation at 380 nm and normalized at 440 nm.

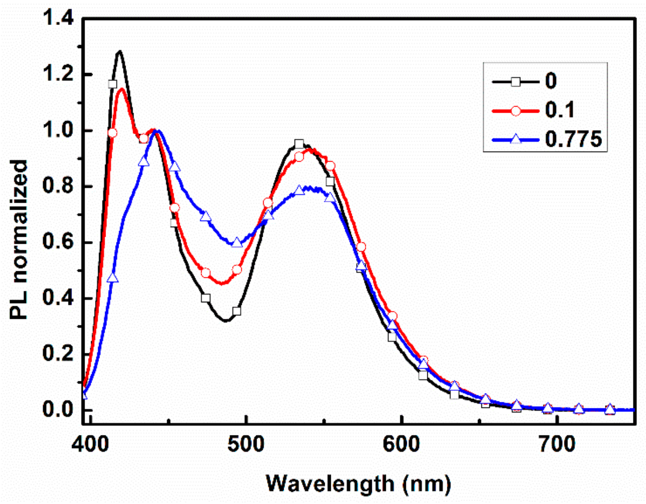

Supplement: Supplementary file 1 [file polymers-09-00227-s001.pdf]
